# Home Manufacture of Formaldehyde for Sterilizing Purposes

**Published:** 1904-05

**Authors:** H. L. Wheeler

**Affiliations:** New York


### HOME MANUFACTURE OF FORMALDEHYDE FOR STERILIZING PURPOSES.[1]

BY DR. H. L. WHEELER, NEW YORK.

The particular use of formaldehyde that I shall mention in this paper is in connection with the sterilizing of instruments. Ever since I have boiled my instruments to make them aseptic I have been looking for a suitable means to produce the same result, and at the same time avoid some of the evil effects of boiling water upon steel and wood, ivory, celluloid, etc. The advent of formaldehyde, or, more accurately, formic aldehyde, as a powerful germ

[1] Read before The New York Institute of Stomatology, January 5, 1904.

destroyer seemed to offer the needed substance; and my object in coming before you this evening is to suggest to you an easy way to manufacture this powerful germicidal gas. Formaldehyde ($CH_2O$) is produced by dry distillation of calcium formate or by the oxidation of methyl alcohol. It is a gas, and is readily absorbed by water, so that a forty per cent. solution is upon the market under the proprietary name of Formalin. For instrument and room disinfection it is prepared either by some dehydrating agent like calcium chloride, or by lamps for burning wood alcohol.

My experience with several of these specially constructed and patented lamps for this purpose having been decidedly unsatisfactory, and an attempt to get the patentee in one case to strive to remedy the difficulty having proved futile, I set about to find something that could be used steadily and regularly without failure in a short period of time. So far as I know, all the lamps sold for this purpose depend upon a platinized asbestos for the agent that will become incandescent and produce the oxidizing process which breaks up the wood alcohol into formic aldehyde ($CH_2O + H_2O$). This platinized asbestos is made by a secret process, and can be purchased by the ounce from any chemical supply house, but it has not proved lasting in the lamps I have used. To remedy its annoying tendency to fail when needed, I adopted the plan, after some experimenting, of taking a small piece of sponge platinum cut thin and holding it just above the wick of any ordinary alcohol lamp by any contrivance one may wish to construct. Light the wick and heat the platinum to incandescence; then extinguish the flame of the lamp and the metal will retain a dull glow and continue to produce formaldehyde as long as there is alcohol in the lamp. I simply bend a piece of German silver wire the desired shape for holding my sponge platinum in place, as is shown by the lamp I will pass around. I have also found that the ordinary lamp used under a chafing-dish, which is filled with asbestos covered with wire netting, works admirably for this purpose, only you must first, after lighting the lamp, hold the piece of metal up where it will become thoroughly heated, then extinguish the flame and drop the platinum upon the wire netting, and you will produce the gas. These lamps can be used in any suitable inclosure, as an oven of tin or copper, a tin box, or a chafing-dish, cutting a hole through the bottom large enough for the burner of the lamp to extend inside the receptacle; and the instruments can be placed in any way desired within the inclosure. The amount of formaldehyde manufactured in a given time depends upon the area of the piece of platinum and the size of the wick. I do not know if this suggestion has ever been published before; if so, I have not seen it, and I give it to you in detail that you may not be prohibited from using it by a patent; for patents seem to be becoming more and more an injury, and are threatening to entirely erase what ethics we possess. It is my desire to call attention to the advantages of publishing, as a matter of record, without securing a patent, those ideas, mechanical and otherwise, which we think may be of advantage to the profession and, through the profession, to humanity.

In order to do this I shall call attention to some examples which will necessitate showing conversely how we have suffered from patents, largely due to a lack of reasonable foresight and a proper regard for ethical standards upon our part. Almost with one accord we subscribe to the funds of the Dental Protective Association to prevent our being mulcted by the Tooth-Crown Company, and yet as an organized body of men we have taken not one step to prevent the same evil coming upon us in the future in the only way it could be prevented,—viz., to promptly deny standing in any of our societies to any member of our profession who becomes so forgetful of his duties as a professional man as to acquire a patent on any implement or material to be used by the profession. We have courted a recurrence of the difficulty by an apparent approval of those who obtain patents, as is evidenced by observing what a large proportion of men invited to appear before our societies are there for the precise purpose of calling attention to some device which they have patented. From a purely financial point of view this policy of encouraging patents and at the same time raising large sums to fight them is such a lack of intelligent self-interest that I cannot conceive of a body of men of less than ordinary ability who would do these things as a plain business proposition. Note how large sums of the Dental Protective Association were spent in fighting the patents on the Donaldson broach, —in the interest of a rival supply house, unless I am misinformed, —and, after proving invalid a patent that had nearly expired, we are still paying the original price of two dollars and fifty cents per dozen for the genuine Donaldson broaches because those made to imitate them are of such a quality that they in no way compete with the original. Place beside this the decrease in the price of dental burs. Dr. William Rollins, of Boston, published a cut of the so-called "revelation" bur, or a similar one, in the *Archives of Dentistry,* January, 1885. He had previous to this tried to persuade a well-known dental supply house to manufacture this bur, but without success; later they were patented by this supply house, and they began their manufacture. Subsequently a rival house began the making of the same bur, and in the attempt to get an injunction upon this latter house the patent was proved invalid, because Dr. Rollins had published a description of the bur prior to the granting of the patent. The ultimate result was we were able to obtain burs at one dollar per dozen which before had cost two dollars and twenty-five cents, all due to one man having the foresight to publish his device without covering it with letters patent; and it did not cost the profession thousands upon thousands of dollars to prove the patent invalid.

I notice, in a late appeal for more funds from the members of the Dental Protective Association, it is stated that we have been protected from paying royalty on inlays by this Association. The author of this statement must be misinformed, or he lacks information; and that it will probably pass as authoritative indicates what a lack of knowledge there appears to be among the members of our calling, due, no doubt, to our habit of being fed pap by the supply houses through their faithful organs the dental journals published by them. It is a matter of record that Dr. Rollins described various methods for making metal moulds for porcelain fillings in a paper read before the Boston Society for the Advancement of Oral Science in June, 1880, while porcelain inlays ground to fit the cavity were used by Dr. Volck and his method published in the *American Journal of Dental Science* for July, 1857. In view of these published records, is it not obvious that patents taken out by Dr. Land in 1887 upon certain methods could not have required any great amount of time or expense to have proved them invalid? I am minded also of the case of the cervical clamp devised by Dr. Woodward and suppressed because a supply house claimed it was an infringement on an instrument already manufactured by them; although this latter instrument is greatly inferior to Dr. Woodward's. Were members of our profession to refuse to take out patents, and to carefully publish for record their inventions, the supply houses could not use a patent on an inferior device as a club to suppress superior instruments in which, in many instances, there is a grave doubt if there be any infringement whatever. The organized corporation has the advantage, as they mostly retain an attorney by the year, part of whose duties is to protect the corporation by shutting off all implements possible that would compete with their own product; and every time a member of our profession takes out a patent and sells it to a manufacturer he is increasing the difficulty of every member of the profession to obtain instruments suited to his individual taste and necessities.

